# Ultrasensitive detection and characterization of molecules with infrared plasmonic metamaterials

**DOI:** 10.1038/srep14327

**Published:** 2015-09-21

**Authors:** Fei Cheng, Xiaodong Yang, Jie Gao

**Affiliations:** 1 Department of Mechanical and Aerospace Engineering, Missouri University of Science and Technology, Rolla, MO 65409, USA

## Abstract

Infrared vibrational spectroscopy is an effective technique which enables the direct probe of molecular fingerprints, and such detection can be further enhanced by the emerging engineered plasmonic metamaterials. Here we experimentally demonstrate ultrasensitive detection and characterization of polymer molecules based on an asymmetric infrared plasmonic metamaterial, and quantitatively analyze the molecule detection sensitivity and molecule-structure interactions. A sharp, non-radiative Fano resonance supported by the plasmonic metamaterial exhibits strongly enhanced near-field, and the resonance frequency is tailored to match the vibrational fingerprint of the target molecule. By utilizing the near-field nature of the plasmonic excitation, significantly enhanced absorption signal of molecules in the infrared spectroscopy are obtained, enabling ultrasensitive detection of only minute quantities of organic molecules. The enhancement of molecular absorption up to 10^5^ fold is obtained, and sensitive detection of molecules at zeptomole levels (corresponding to a few tens of molecules within a unit cell) is achieved with high signal-to-noise ratio in our experiment. The demonstrated infrared plasmonic metamaterial sensing platform offers great potential for improving the specificity and sensitivity of label-free, biochemical detection.

Plasmonic metamaterials and metasurfaces have exhibited a variety of exotic optical properties that go beyond those achievable using natural materials, such as negative refractive index^1^,^2^, indefinite permittivity^3^,^4^,^5^ and nonlinear polarization rotation by chiral metadevices^6^. Such extraordinary optical properties have enabled many unprecedented applications, ranging from perfect lenses^7^ to invisible cloaking^8^, perfect absorbing^9^,^10^ to lasing spaser^11^,^12^. Among all the promising applications, biochemical sensing techniques such as surface-enhanced Raman scattering (SERS)^13^ and surface-enhanced infrared absorption (SEIRA)^14^ have been making significant progresses in recent years. As compared to earlier SEIRA studies^15^,^16^ involving uncontrolled, chemically prepared or roughened metal surfaces, the emerging delicately engineered plasmonic metamaterials serve as a more powerful biochemical sensing platform, based on either collective excitation of periodic nanostructures^17^,^18^,^19^ or local resonances of single metamolecules^10^,^20^. Both kinds of plasmonic metamaterials are of special interest from the standpoint of quantitative biosensing with good specificity because large local near-field enhancement can be provided exactly at the vibrational fingerprints of biomolecules in the mid-infrared spectroscopy (typically in the 3–6 *μ*m wavelength range)^21^. However, for quite a few plasmonic enhanced biosensing devices, achieving the desired hot spots exhibiting strongly enhanced near-field typically requires nanometer-sized air gap (<10 nm) between plasmonic resonators^22^. Although a gap size smaller than 5 nm can be realized by delicate microfabrication processes^23^,^24^, the exquisite control of plasmonic nanostructures within the sub-10 nm regime is still very challenging, especially for traditional microfabrication methods such as electron beam lithography (EBL) limited by the proximity effect or focused ion beams (FIB) milling affected by the second deposition processes.

Recently, alternative strategies relying on the coupling of surface plasmon polaritons or localized surface plasmons in metallic nanostructures are emerging, e.g., Fano resonances^25^,^26^,^27^,^28^,^29^,^30^,^31^,^32^ and analogue of electromagnetically induced transparency (EIT)^33^,^34^,^35^,^36^. Fano resonances originate from the plasmonic hybridization^37^ between two electromagnetic eigenmodes within a nanostructure, which are often distinguished as bright and dark modes that possess intrinsically different radiative losses. Though the dark mode cannot be directly excited by the incident radiation, the plasmonic hybridization transfers optical activity to the dark mode and yields sharp asymmetric resonances with high quality factors^26^. Another important reason why Fano resonances have been drawing more attentions recently is that less-complicated fabrication processes are required compared to the delicate biosensing devices mentioned above. For example, a simple Fano-resonator has been proposed by Wu *et al.* recently for the detection and characterization of ultrathin multiprotein layers^38^, showing attractive advantage of asymmetric metamaterials for biosensing applications over the symmetric delicate counterparts.

Here we experimentally demonstrate the ultrasensitive detection and quantitative characterization of poly(methyl methacrylate) (PMMA) molecules utilizing specially designed asymmetric Babinet-inverted Fano-resonant plasmonic metamaterials (FRPMs). As a complementary structure of nanoantennas normally used for biodetection^17^,^38^,^39^,^40^,^41^, the cut-out nanostructures in a continuous metal film can be readily realized by focused-ion-beam writing or nanoimprint lithography^41^, and the sensing medium easily fills the voids in the film and thus facilitates the detection of the target biomolecules^34^. The polarization-dependent spectroscopic properties of the asymmetric FRPMs enable the accurate experimental determination of the spectral position of the Fano resonance. As a proof-of-concept demonstration for the FRPMs biosensing platform, the superior sensing capabilities are tested by loading well-defined thin polymer layers on a series of fabricated FRPMs. The non-radiative Fano resonance is designed to match the carbonyl bond absorption fingerprint at 1733 cm^−1^ (~52 THz). Large spectral and spatial overlap between the strongly confined near-field of the plasmonic mode and molecular vibrational absorption dramatically boosts transduction of molecular structural properties into detectable infrared signals, enabling the detection of minute amount of molecules on the plasmonic platforms.

## Results

### Mid-infrared responses of FRPMs

**Figure 1:**
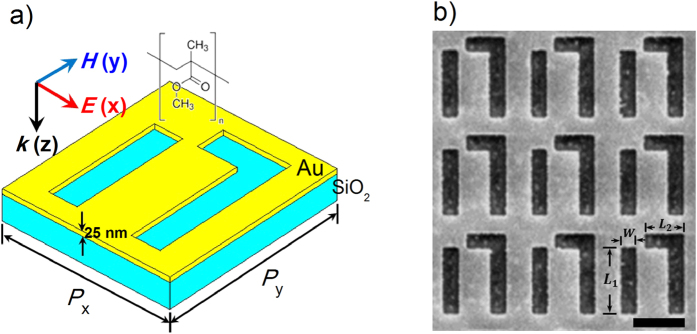
Infrared plasmonic metamaterials fabricated on gold films. (**a**) Schematic of a unit cell of the designed infrared plasmonic metamaterial and the incident light polarization configuration. (**b**) SEM image of a selected nanostructure fabricated on a glass substrate. 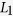 = 1.3 *μ*m, 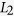 = 0.48 *μ*m, *w* = 0.3 *μ*m and periodicities along *x* and *y* directions are 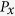 = 1.7 *μ*m, 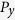 = 1.9 *μ*m, respectively. Scale bar: 1 *μ*m.

**Figure 2:**
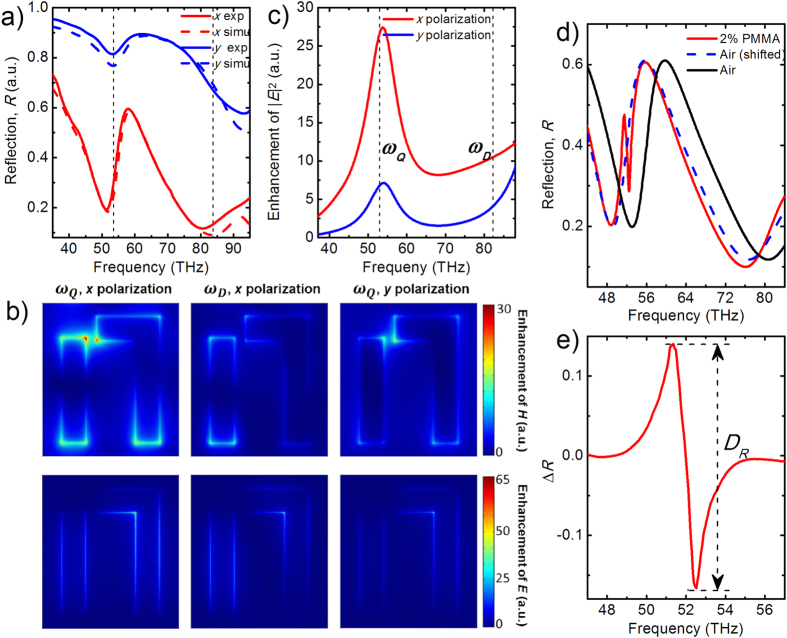
Optical characterizations of a representative FRPM. (**a**) Measured (solid curves) and simulated (dashed curves) polarized reflection spectra. (**b**) Field profiles and enhancement of |***E***| and |***H***| (color bar) calculated on a plane 5 nm above the metamaterial at the resonant frequencies marked in panel a. (**c**) Calculated enhancement spectra of electric field intensity for the FRPM shown in panel b. Field intensities are averaged within a 20-nm-thick layer above the metamaterial. (**d**) Reflectance spectrum from a typical FRPM sample before (*R*_*bare*_ black solid) and after (*R*_*func*_ red solid) the coating of PMMA molecule layer (from solution with 2% solid content). The blue dashed curve shows the frequency-shifted spectrum considering the red-shift effect of polymer with nondispersive refractive index. (**e**) Refl**e**ctance difference spectrum (∆*R* = *R*_*func*_ − *R*_*bare*_) with the defined signal strength (*D*_*R*_) indicated.

Figure 1 illustrates the schematic and scanning electron microscopy (SEM) image of a typical asymmetric FRPM fabricated on a 25 nm thick gold film by focused ion beam (FIB, Helios Nanolab 600), revealing nanovoids with well-defined square corners and minute amount of edge roughness. Each unit cell consists of two parallel cut-out slot antennas along the *y* axis, in which the right one is end-connected to a perpendicular shorter slot antenna. By breaking the spatial inversion symmetries of the unit cell in the structure plane, Fano interference is enabled for the *x*-polarized incident light for the Babinet-inverted FRPM^42^,^43^. The mid-infrared response of the FRPMs at normal incidence is characterized by a Fourier-transform infrared (FTIR) spectrometer for both *x*- and *y*-polarized incidence, as shown in Fig. 2(a). In the *x*-polarized reflection spectrum (red solid), the broad symmetric Lorentzian resonance at 83 THz represents the slot *dipole* resonance (bright mode *ω*_*D*_), and the narrow asymmetric resonance around 53 THz corresponds to the Fano interferences between the slot *dipole* and two-slot *quadrupole* resonance (dark mode). The resonant frequency (*ω*_*Q*_) of the latter mode can be determined from *y*-polarized reflection spectrum (blue solid) where the dark mode is excited alone without the interference with the bright mode^38^,^44^.

To get a better understanding of the underlying physics, numerical simulations using the finite-element method (COMSOL Multiphysics) are carried out to obtain the reflection spectra, electromagnetic field distributions and field enhancement spectra of the FRPMs. Here the permittivity of bulk gold in the mid-infrared is described by the Drude model (detailed in Supplementary Information) and the permittivity of glass substrate is taken from the refractive index database^45^. The calculated reflection spectra for the FRPM shown in Fig. 1(b) are plotted as dashed curves in Fig. 2(a) for both polarizations, showing good agreement with the experimental data. The small discrepancies between them are likely due to the fabrication tolerances in the experiment such as the inhomogeneity in the corners of the slots. The cross sectional views of the magnetic field distributions for both bright and dark modes under *x*- and *y*-polarized excitations are shown in the top panel of Fig. 2(b). The magnetic *quadrupole* excitation at *ω*_*Q*_ exhibits significant field concentration near the end and the gap of the slots with typical Fano interference field pattern^38^. The well-confined, enhanced near-field profile and suppressed radiation damping of the *quadrupole* mode are reflected as a narrowing of the far-field spectral response. No interference effects are observed, however, for the slot *dipole* resonance at *ω*_*D*_. On the other hand, the electric field distributions for both modes (bottom panel of Fig. 2(b)) show distinct differences from those observed in the previously reported positive nanoantennas^17^,^38^. For the Babinet-inverted FRPMs, the electric field confines mainly on edges perpendicular to the incident polarization. The field maximum locates at the inner corner of the right slot with a local field intensity enhancement in the range of 10^3^ ∼ 10^4^ (detailed in Fig. S4(a)). The maximum field intensities are observed to be much weaker for the superradiant *dipole* and *y*-polarized subradiant *quadrupole* mode. The calculated averaged intensity enhancement spectrum (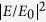 averaged over a hypothetical 20-nm-thick layer above the metamaterial) shown in Fig. 2(c) illustrates clearly that a maximum intensity enhancement is obtained at the Fano resonance for x polarization, which holds promise for the ultrahigh sensing sensitivity.

In order to demonstrate the molecule sensing capability of the FRPMs, a thin layer of poly(methyl methacrylate) (PMMA) molecules dissolved in anisole (950A-2, Microchem) is deposited by spin coating on the fabricated metamaterials. The carbonyl bonds in PMMA molecules (~1733 cm^−1^ or 52 THz, *ω*_*m*_) possess large intrinsic dipole moment, and uniform PMMA layers can be formed with accurately controlled thicknesses by varying the molecule concentrations in the anisole solvent. A series of FRPMs with uniformly scaled dimensions are designed and fabricated to have tunable Fano resonance frequencies sweeping across the stretching absorption band of PMMA. A good match of the plasmonic resonance to the molecular absorption feature^39^ is crucial for the detection of target molecules, and as demonstrated below, strengthened infrared signal is observed when the plasmonic mode is tuned on-resonant with the molecular vibrational absorption and the signal is gradually deceased as the plasmonic resonance is detuned away from the absorption line. The measured reflection spectrum of a typical FRPM spin-coated with a 100 nm PMMA layer (red solid) is shown in Fig. 2(d), together with that of the bare structure exposed in the air (black solid). The sharp spike feature observed around 52 THz is the result of the enhanced infrared absorption signal due to the strong molecule-FRPM interaction, and the reflectance difference spectrum (∆*R* = *R*_*func*_ − *R*_*bare*_) in Fig. 2(e) is used to better illustrate and analyze the spectral response of FRPMs to PMMA molecules. Redshift of the reflection spectrum^10^,^17^ due to the surrounding refractive index (*n*_PMMA_ ≈ 1.5) aside from any molecule-FRPM interaction effect has been taken into account (detailed in Supplementary Information), and a shifted spectrum of uncoated structure (blue dashed in Fig. 2(d)) is used for *R*_*bare*_ in the subtraction. The signal strength *D*_*R*_ indicated in Fig. 2(e) is defined as the contrast between the maximum and minimum of the ∆*R* spectrum around the spike feature^39^, where the ∆*R* spectrum exhibits an asymmetric lineshape rather than the typical symmetric Lorentzian lineshape for the molecule absorption. Previous observations of asymmetric Fano-like absorption signal in metal-island films^46^ and nanoantenna systems^10^,^17^,^39^,^47^ originate from the coupling between the *dipole* plasmonic resonance and the spectrally narrow molecule absorption. However, in this work, the plasmonic resonance supported by the FRPMs is itself a *quadrupole* mode with Fano-like sharp linewidth and possesses much larger near-field intensity enhancement as compared to the *dipole* resonance. As a result, the molecule-FRPM interaction is significantly enhanced in the double-Fano system and thus substantial signal strength *D*_*R*_ well above the noise level can be obtained. Control measurements are also performed on bare gold film as shown in Fig. S3. Compared to the large signal strength *D*_*R*_ ≥ 0.3 observed from the FRPM coated with 2% solid content PMMA (Fig. 2(e)), a much weaker strength ~0.01 is observed on bare gold film loaded with the same amount of molecules (signal strength <0.01 is observed for 1% solid content PMMA), and spectrum features are even below the noise level for control measurements on the silica substrate.

### Quantitative analysis of FRPM enhanced molecular sensing

**Figure 3:**
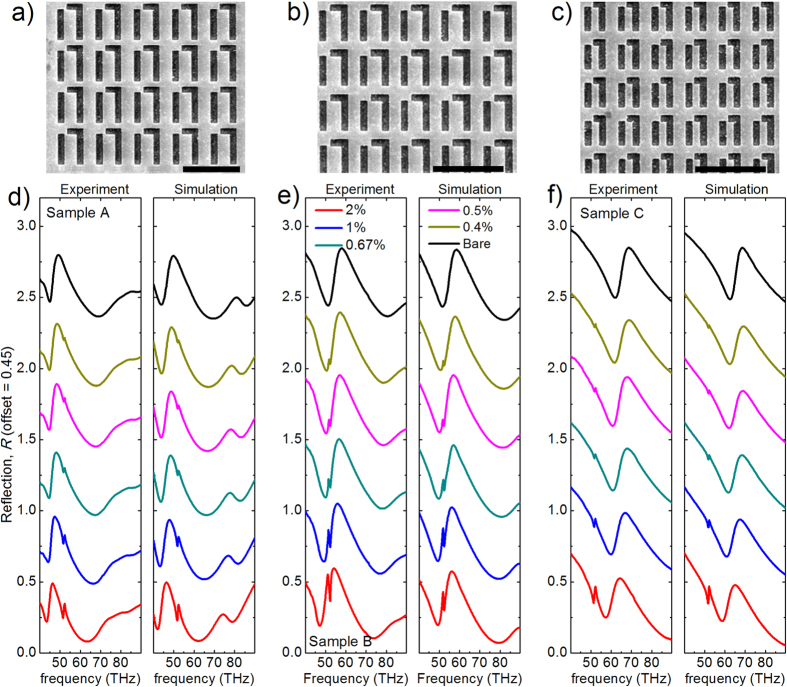
Measured and calculated reflection spectra of three selected FRPM samples loaded with PMMA molecules at different concentrations. (**a–c**) SEM images of three selected FRPM samples A, B and C. Scale bar: 3 *μ*m. (**d–f**) Experimental and simulation reflectance spectra of the three selected samples in (**a–c**) loaded with diluted PMMA molecules in anisole solution at different concentration ***c*** = 2%, 1%, 0.67%, 0.5% and 0.4%.

**Figure 4:**
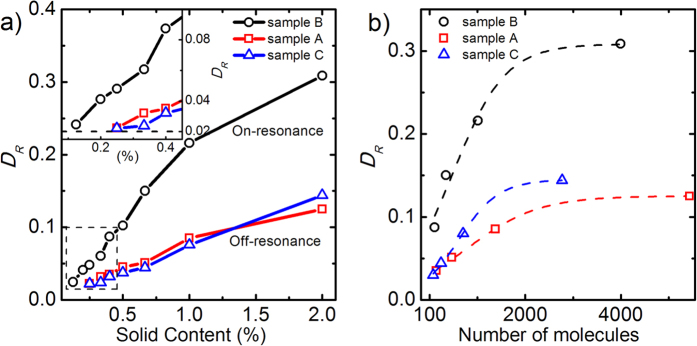
Comparison of the measured signal strengths from three FRPM samples for PMMA molecule sensing. (**a**) Dependence of the measured signal strength on the solid content of PMMA solution obtained from sample A–C in Fig. 3. The inset shows an amplified view of signal strength at extreme low solid content. (**b**) Signal strength *D*_*R*_ as a function of molecule numbers within each unit cell for the three sets of FRPMs. Dashed lines are guides to the eye.

In order to quantitatively and systematically evaluate the sensing ability of FRPMs for detecting the PMMA molecules, three sets of metamaterials with different geometric dimensions are designed and fabricated, and the SEM images are shown in Fig. 3(a−c). The measured and calculated reflection spectra of metamaterials loaded by a thin PMMA layer with progressively decreasing molecules (spun-cast from the diluted PMMA solutions with different concentrations ***c**,* from 2% to about 0.4% solid content in anisole solvent) are shown in Fig. 3(d−a). The X-ray reflectivity measurement is carried out here to confirm the uniformity and film thicknesses with different molecular concentrations (Fig. S1(a)). The PMMA layer is modeled by a Lorentz oscillator material^48^ in calculation (detailed in Supplementary Information) and excellent agreement between experimental and numerical results is obtained. The presence of PMMA layer changes the dielectric environment of the FRPMs and leads to a frequency shift (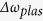) of the plasmonic mode. Here we assume that the permittivity tensor of PMMA molecules is isotropic at the specific absorption frequency. When the polymer thickness *h* is smaller than the near-field decay length of the plasmonic mode (detailed in Supplementary Information), the frequency shift scales linearly with *h* as 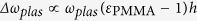. As illustrated in Fig. S1(b), our experimental observation is in accordance with this relationship, and the frequency shifts of all three samples increase linearly with polymer thickness (*h* ≤ 40 nm) with almost identical slopes. By comparing the measured reflection spectra of the respective samples, we note that a distinct molecular absorption signature is observed around 52 THz when the frequency of Fano resonance is tailored to match the frequency of the carbonyl bond of PMMA molecules (on-resonance case, sample B in Fig. 3(e)). While when the geometries of unit cell are scaled to tune the Fano resonance away from the molecular absorption (off-resonance cases, sample A and C in Fig. 3(d,f), respectively), much weaker absorption signals are observed as compared to the on-resonance case. Quantitative analyses of the measured signal strength *D*_*R*_ are presented in Fig. 4(a). Significantly enhanced signal strength (*D*_*R*_ ≈ 0.31 at ***c*** = 2%), corresponding to the strongest interaction between PMMA molecules and FRPMs, is observed for sample B which combines the strengths of narrow line-width of the Fano resonance (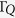) and perfect spectral overlap with the molecule absorption (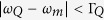). However, when the Fano resonance is tuned away from the absorption line (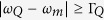), much smaller signal strength (*D*_*R*_ ≈ 0.12 (0.14) for sample A (C) at ***c*** = 2%) is detected due to the weak molecule-FRPM interaction at the off-resonance condition. As the solid content of molecules is progressively diminished from 2% to 0.25%, the signal strength observed for sample A and C decreases to less than 0.02 and approaches the noise level of our measurement. Contrastingly, signal strength larger than 0.04 is observed for sample B. Especially, when the solid content of PMMA molecules further decreases from 0.2% down to 0.125%, the detectable signal strength is only observable from sample B (as shown in the inset of Fig. 4(a) and Fig. S2), which demonstrates the ultrahigh sensing sensitivity of the on-resonance FRPMs.

The controllable PMMA film thickness ranging from hundreds of nanometers to a dozen nanometers provides an opportunity to investigate the ultimate detection limit of molecule numbers using the FRPMs. According to the X-ray reflectivity measurements, the thickness of a diluted PMMA layer with 0.4% solid content is 12.8 nm (Fig. S1(a)), corresponding to about 100 molecules in each unit cell (detailed in Supplementary Information). As a result, the measured signal strength (*D*_*R*_ ≈ 0.09 for sample B) in the case of 0.4% solid content is obtained from about 77400 molecules or 130 zeptomoles for the entire array (50 × 50 *μ*m^2^). Absorption signals from further diluted PMMA solution (molecule amount down to tens of zeptomoles) have also been observed as shown in Fig. S2(b). Figure 4(b) shows the measured signal strength as a function of the number of molecules within each unit cell for the three FRPMs samples. Rapid initial increase of the signal strength follows an almost linear dependence of the number of molecules when the solid content is smaller than 1% (~1 × 10^3^ or less molecules within each unit cell), but the signal strength subsequently appears to saturate at larger number of molecules when the film thickness is more than 40 nm. The observed saturation behavior of the measured signal strengths can be attributed to the evanescent plasmonic near-fields decaying away from the FRPMs surface, which was also observed in nanorod arrays loaded with varying protein film thickness^17^. Confirmed by our FEM simulations in Fig. S4(d), the near-field intensity of FRPMs (at position A) decays rapidly along *z* axis to a value of 1/e^2^ of the maximum within about 30 nm, which validates the near-field nature of enhancement mechanism of the FRPM-based molecular detection. The demonstrated detection sensitivity (zeptomole level) is quite impressive as compared to previous works, and it is also important to note that the estimation of detectable amount of molecules is conservative because only the portion of molecules that experience the near-field interaction with the FRPM exhibit the enhanced infrared absorption and contribute to the measured enhanced signals. According to the near-field intensity distribution of the Fano resonance in Fig. S4, a higher sensitivity can be achieved if only the active sensing areas (around point A in Fig. S4) are taken into account.

Furthermore, in order to provide more insights into the sensing mechanism, the absorption enhancement factor of the FRPMs is estimated from the experimental and numerical results. At first, the measured signal strength from PMMA molecules on the FRPM structures (*D*_*R*_ ≈ 0.22 from sample B with solid content *c* = 1%) is compared to the signal strength from the same amount of molecules on a reference bare gold film (*D*_*R*_ ≈ 0.007). Then an enhancement by the mirror-dipole effect (a factor of 2) from the gold film^49^ and the screening factor 1/(1 + *n*_*s*_)^39^,^50^ from the sample substrate have to be considered (detailed in Supplementary Information). In addition, the electric field intensity (|***E***|^2^) distribution of the Fano resonance is plotted in Fig. S4. The field intensity concentrates at the inner corner (point A) within the unit cell, and the field intensity attenuates drastically along the *x* and *y* directions with spatial extents of 60 nm (*l*_x_) and 90 nm (*l*_y_) respectively. The active area can be estimated by (*l*_x_ + *l*_y_)·*h*_Au_, where *h*_Au_ is the thickness of Au film. Taking into account all the above mentioned factors, the enhancement factor of the infrared absorption of molecules that interact with the FRPM is estimated to be *D*_*R*_(FRPM)/*D*_*R*_(Au)·2·(1 + *n*_*s*_)·*A*_0_/(*l*_x_ + *l*_y_)·*h*_Au_ ≈ 163,000, where *A*_0_ is the size of unit cell. Therefore, the demonstrated FRPM provides an enhancement factor of the molecular absorption signal larger than 10^5^, which is remarkable compared to the reported SEIRA results^17^,^39^.

### Phenomenological model describing the molecule-FRPM interaction

To examine the enhanced molecule-FRPM interaction strengths in the presence of the *dipole* resonant mode, a mechanical model consisting of coupled damped harmonic oscillators^51^,^52^,^53^ is developed to describe the spectral response of the FRPMs. The radiative slot *dipole* is represented by one oscillator with resonance frequency *ω*_*d*_ and damping rate *γ*_*d*_, and the nonradiative two-slot *quadrupole* is represented by another oscillator with resonance frequency *ω*_*q*_ and damping rate *γ*_*q.*_ The coupling coefficient between the two oscillators is *σ*_*dq,*_ and the externally applied harmonic driving force is *f·e*^*iwt*^. Taking the absorption of molecules on the FRPMs into account, a third harmonic oscillator with resonance frequency *ω*_*m*_ and damping rate *γ*_*m*_ is introduced to form a three-oscillator coupled system^32^,^54^. The coupling coefficient between the molecule and the *dipole* (*quadrupole)* mode is *σ*_*dm*_ (*σ*_*qm*_). The motion equations of three oscillators can be written as:


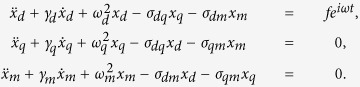


**Figure 5:**
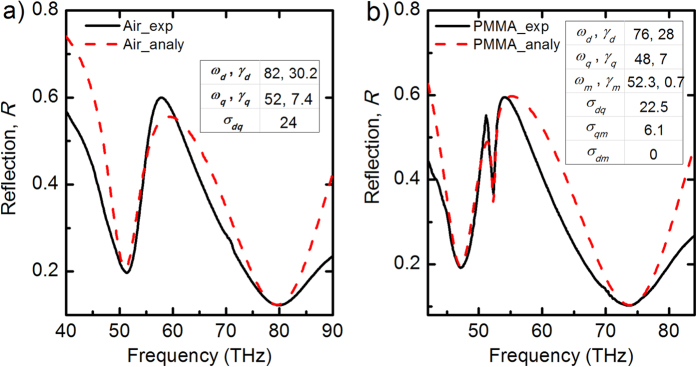
Analysis of the molecule-FRPM interactions with the coupled harmonic oscillator model. (**a**) Comp**a**rison between the measured reflection spectrum of bare sample B (black solid) and the calculated reflection spectrum from the coupled harmonic oscillator model (red dashed). (**b**) Comparison between the measured reflection spectrum of functionalized sample B coated by PMMA molecules (black solid) and the calculated reflection spectrum from the modeling (red dashed). Extracted fitting parameters of the resonance frequencies (in the unit of THz), the damping rates (in the unit of THz) and the coupling strengths (in the unit of THz^2^) are presented in the insets.

The displacements *x*_*d*_, *x*_*q*_ and *x*_*m*_ of the oscillators are harmonic with *x*_*d, q, m*_ = *c*_*d, q, m*_·*e*^*iwt*^ where the amplitudes *c*_*d, q, m*_ can be calculated analytically. We obtain the reflection spectrum by summing the square of three oscillation amplitudes and subtract them from unity, which represents light scattering efficiency of the system. The reflection spectra of sample B obtained from the coupled-oscillator modeling are shown in Fig. 5 (red dashed) for the bare and functionalized FRPMs (results of sample A and C are shown in Fig. S5), demonstrating a reasonable agreement with the experimental results (black solid). The resonance frequencies of the plasmonic modes and molecule absorption are taken directly from the experiment values. The damping rates and the coupling coefficients can be extracted from the oscillator model. We note that the damping rates of the Fano resonance and molecular absorption are relatively smaller (*γ*_*q*_ = 7 THz, *γ*_*m*_ = 0.7 THz) compared to that of the *dipole* resonance (*γ*_*d*_ = 28 THz), which capture our observations in the experiment well. The extracted coupling coefficients from the measured three samples (see inset of Fig. 5 and Table 1 in the Supplementary Information) reveal that a strong interaction strength between the plasmonic resonance and molecular absorption band (well overlapped with each other) play a key role in achieving an ultrahigh sensitivity, compared to that of a much weaker molecule-plasmonic interaction with a negligible coupling coefficient.

## Discussion

In conclusionwe have designed and demonstrated ultrasensitive molecule detection and characterization based on non-radiative FRPMs exhibiting strongly confined near-field modes with sharp spectral features. The plasmonic mode can be engineered to match the absorption fingerprint of the target molecules and up to 10^5^-fold enhancement of absorption signal is obtained. Detection of zeptomole levels of molecules has been demonstrated in experiment with high signal to noise ratio, corresponding to only few tens of molecules in each unit cell. A phenomenological model is also introduced to provide a better understanding of the underlying mode interaction mechanism. This work opens a new route for metamaterial applications toward biochemical sensing of minute mass concentrations as well as selective detecting of biomolecules at a nanometer scale.

## Methods

### Metamaterial fabrication

In brief, the gold film is deposited onto a silica substrate using electron-beam evaporation method (evaporation rate 0.5 Å/s). The asymmetric Fano resonant plasmonic metamaterials (FRPMs) are fabricated via focused ion beams, carried out in a FEI Helios Nanolab 600 DualBeam microscope system with the focused beam of gallium ions of the current of 9.7 pA and the energy of 30 eV. Each sample has a 50 × 50 *μ*m^2^ milled area sufficient for the optical reflection measurements.

### PMMA layer preparation

Different concentrations of poly(methyl methacrylate) (PMMA) molecules, a commonly used positive electron-beam resist, are used as model analyte in our experiment. For the largest concentration, a thin layer of PMMA (950-A2, 2% solid content in anisole, Michrochem) is spin-coated on top of the metamaterials at 2000 rpm. PMMA is chosen in the present work due to the accurate control of the uniform thickness obtainable via control of the spin speed and molecule concentration used. Then molecule concentration is diluted progressively in anisole and the diluted polymer solution is spun onto nanostructures. The dielectric function of the polymer can be modeled as *ε*_dilute_ = *f·ε*_PMMA_ + (1−*f*)·*ε*_anisole_ in numerical simulations, where *ε*_PMMA_ and *ε*_anisole_ are permittivity of readily obtained PMMA (950-A2) and anisole and *f* is the filling ratio of the PMMA. The thicknesses of diluted polymers with different concentrations are determined through X-ray reflectivity (Philips X'Pert-MRD) measurement and the respective thicknesses of different concentrations are shown in Fig. S1(a).

### FTIR measurements

The reflection spectra of the FRPMs are recorded using a Fourier transform infrared (FTIR) spectrometer (Thermo Scientific, Nicolet 4700). Reflected signals are collected with a 0.4 NA Compensation objective and recorded by a liquid-nitrogen-cooled mercury cadmiumtelluride (MCT) detector. All the spectra are recorded with a resolution of 4 cm^−1^ and 512 scans. The measurements are normalized with respect to a silver coated mirror (THORLABS). An IR polarizer (ZnSe, THORLABS) is used to polarize the incident electromagnetic field perpendicular or parallel to the slot antennas. The reflectance difference spectra displayed in Fig. 2(d) and Fig. S2 are calculated through ∆*R* = *R*_*func*_ − *R*_*bare*_, where *R*_*func*_ and *R*_*bare*_ are the reflectance spectra of PMMA-coated FRPMs and that of frequency-shifted bare FRPMs, respectively. The frequency shift is performed via a transformation *R*_*bare*_ = *R*_0_ (*ω*_0_) → *R*_0_ (*ω*_0_ + ∆*ω*) to coincide the maximum of two spectra as illustrated in Fig. 2(d), where *R*_0_ (*ω*_0_) is the original reflectance of a bare structure and ∆*ω* is the frequency shift due to the presence of PMMA layers with refractive index *n*_PMMA_ ≈ 1.5. The absorption signal strength is defined as difference between maximum and minimum of the ∆*R* spectra around the absorption line of carbonyl bond stretching.

### Numerical simulations

Finite element method (FEM) simulations are performed to obtain the optical reflection spectra and field distributions using the software (COMSOL Multiphysics). In the simulations, periodic boundary conditions are employed along the *x* and *y* axes to account for the periodic arrangement of the unit cells. Perfectly matched layers (PMLs) surrounded by scattering boundary condition faces are utilized along the propagation direction (perpendicular to the planar metamaterial) to avoid multiple reflections due to geometry truncation. The permittivity of bulk gold in the mid-infrared is described by the Drude model 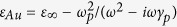, where the background dielectric constant is *ε*_∞_ = 1, the plasma frequency *ω*_*p*_ = 1.37 × 10^16^ rad/s and the damping constant *γ*_*p*_ = 4.08 × 10^13^ rad/s. Due to the surface scattering and grain boundary effects in thin films, the damping constant of gold film in our simulation is taken to be three times that of bulk gold in order to match the experimental results. Meanwhile, to take the molecular absorption effect into account, here the PMMA layer is modeled as a Lorentz oscillator material described by 
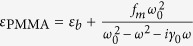
, where *ε*_*b*_ = 2.2 is the background relative permittivity of PMMA, *f*_*m*_ is the reduced oscillator strength, *ω*_0_ = 3.269 × 10^14^ rad/s is the Lorentz resonance frequency of PMMA molecules and *γ*_0_ is the Lorentz damping rate. In our simulation, the values of *f* and *γ*_0_ are appropriately chosen to provide a close match with the experimental measurements. The experimentally observed reflection spectra with the molecule vibrational absorption signals can be numerically reproduced as shown in Fig. 3.

## Additional Information

**How to cite this article**: Cheng, F. *et al.* Ultrasensitive detection and characterization of molecules with infrared plasmonic metamaterials. *Sci. Rep.* **5**, 14327; doi: 10.1038/srep14327 (2015).

## Supplementary Material

Supplementary Information
