# Supplementary material for: Ultrasensitive detection and characterization of molecules with infrared plasmonic metamaterials
Source: Sci Rep. 2015 Sep 21;5:14327. doi: 10.1038/srep14327 (PMC4585698; doi:10.1038/srep14327)
Supplement: Supplementary Information [file srep14327-s1.pdf]

Supplementary Information

Ultrasensitive detection and characterization of molecules with infrared plasmonic metamaterials

Fei Cheng, Xiaodong Yang, and Jie Gao[*]

Department of Mechanical and Aerospace Engineering, Missouri University of Science and Technology, Rolla, MO 65409, USA

[*]Corresponding Email: gaojie@mst.edu

Frequency shift

The detection and characterization of target molecules can be accomplished by measuring both the reflectance difference spectrum and the frequency shift ($\Delta\omega_{plas}$) of Fano resonance, because the PMMA molecules loaded on FFRPMs change the local dielectric environment and the frequency of Fano resonance will be shifted accordingly. Due to the small perturbation of the electromagnetic energy in the dielectric layer, the frequency shift of Fano resonance can be approximately given by[1-4]

$$\frac{\Delta\omega_{plas}}{\omega_{plas}} = -\frac{1}{2}\frac{\int_0^h \vec{E}(\mathrm{r})\cdot(\hat{\varepsilon}-1)\cdot\vec{E}(\mathrm{r})d\mathrm{r}}{\int_0^\infty E^2(\mathrm{r})d\mathrm{r}}$$

where $\vec{E}(\mathrm{r})$ is the near field of the plasmonic mode, $\hat{\varepsilon}$ is the dielectric constant tensor of the PMMA molecules, and h is the PMMA layer thickness. The absolute value of the shift is shown to depend on the overlap between the electric field distribution of the specific surface plasmon mode and the molecular layer[4]. As verified by our numerical simulations, the electric field is predominantly normal to the metal surface and well confined within a thin layer with a thickness of a few tens of nanometers (see Figure S4d). Meanwhile, since the permittivity of PMMA molecules is almost direction-independent at the specific carbonyl bond absorption frequency, the frequency shift calculated from the above simplifies to $\Delta\omega_{plas} = c\omega_{plas}(\varepsilon_{\mathrm{PMMA}}-1)h$ where ***c*** is a constant.

Enhancement factor estimation

A fairly accurate estimation of the enhancement factor of FRPMs can be performed as follows. At first, the measured signal strength D_R(FRPM) ~ 21.62% from sample B coated with PMMA molecules (1% solid content) is compared to the signal strength from the reference substrate

coated with the same amount of molecules. Since the signal from molecules onsilica substrate is below the noise level of the experimental setup, we take a bare gold film as the reference substrate with D_R(Au) ~ 0.55%. As compared to bare silica substrate, the absorbance signal on Au film is already enhanced by the mirror-dipole effect (a factor of 2)[5]. Besides, a substrate "screening" effect which decreases the structural-molecular coupling signal by the factor $1/(1 + n_s)$ ($n_s = 1.4$) needs to be taken into account[6]. In addition, the observed enhanced signal in the experiment is contributed by the molecules within the detection volume at the close vicinity of the plasmonic nanostructure. Due to the fact that the enhancement of infrared vibrational absorption scales with electric field intensity ($|\boldsymbol{E}|^2$) of the local field[7,8], the field intensity distribution 1 nm above the FRPMs at the Fano resonance is examined in Figure S4. The field intensity concentrated at the corner hot spot (denoted by A) is much larger than the intensities at other places within the unit cell with a size of $A_0 = 1.7 \times 1.9\ \mu\text{m}^2$. Along the two dashed lines parallel to x and y directions (Figure S4a), the field intensity decreases drastically to $1/\text{e}^2$ magnitude of maximum in the range of 60 nm (l_x) and 90 nm (l_y), respectively. The active area with intense near-fields is therefore approximated as $(l_\text{x}+l_\text{y})\cdot h_\text{Au}$, where h_Au is the metal thickness. Taking into account all the above-mentioned factors, the enhancement factor of the FRPMs enhanced infrared absorption is estimated to be $D_R(\text{FRPM})/D_R(\text{Au})\cdot 2\cdot(1 + n_s)\cdot A_0/(l_\text{x}+l_\text{y})\cdot h_\text{Au} \approx$ 163,000 in our experiment.

PMMA molecule detection sensitivity

To evaluate the detection sensitivity of the FRPMs, the number of PMMA moleculesin each unit cell of sample B ($A_0 = 1.7 \times 1.9\ \mu\text{m}^2$) and the entire array ($A = 50\ \mu\text{m}^2$, 774 unit cells) will be estimated. Given that molecular weight of poly(methyl methacrylate) and anisole is 950,000 and

108.14, and the density of anisole (anhydrous, 99.7%, Sigma-Aldrich) and PMMA 950 (2% solid content in anisole) is 0.995 g/cm^3 and 0.975 g/cm^3, respectively, the volume filling ratio of PMMA molecules in a spin-coated monolayer (h = 100 nm) is about 3.9% if the molecular chemical binding effect is neglected, about 4000 molecules exist in one unit cell. As the polymer is diluted by anisole step by step to $\boldsymbol{c}$ = 0.67% (h = 25.4 nm) and $\boldsymbol{c}$ = 0.4% (h = 12.8 nm), the volume filling ratio of PMMA molecules decreases to 1.3% and 8‰, corresponding to 340 and 100 molecules in each unit cell. As a result, the measured absorption signal strength ($D_R \approx 8.7\%$) in the case of $\boldsymbol{c}$ = 0.4% is obtained from about 77500 PMMA molecules for the entire array, corresponding to about 130 zeptomoles. The above estimation is conservative because only the molecules that experience strong near-field interaction with FRPMs (near the inner corner point A in Figure S4) exhibit the enhanced infrared absorption and contribute to the enhanced measurement signals. In this way, the FRPMs enhanced sensing sensitivity already achieved in our measurement can be further improved when only the effective area is taken into account.

Supplementary Figures

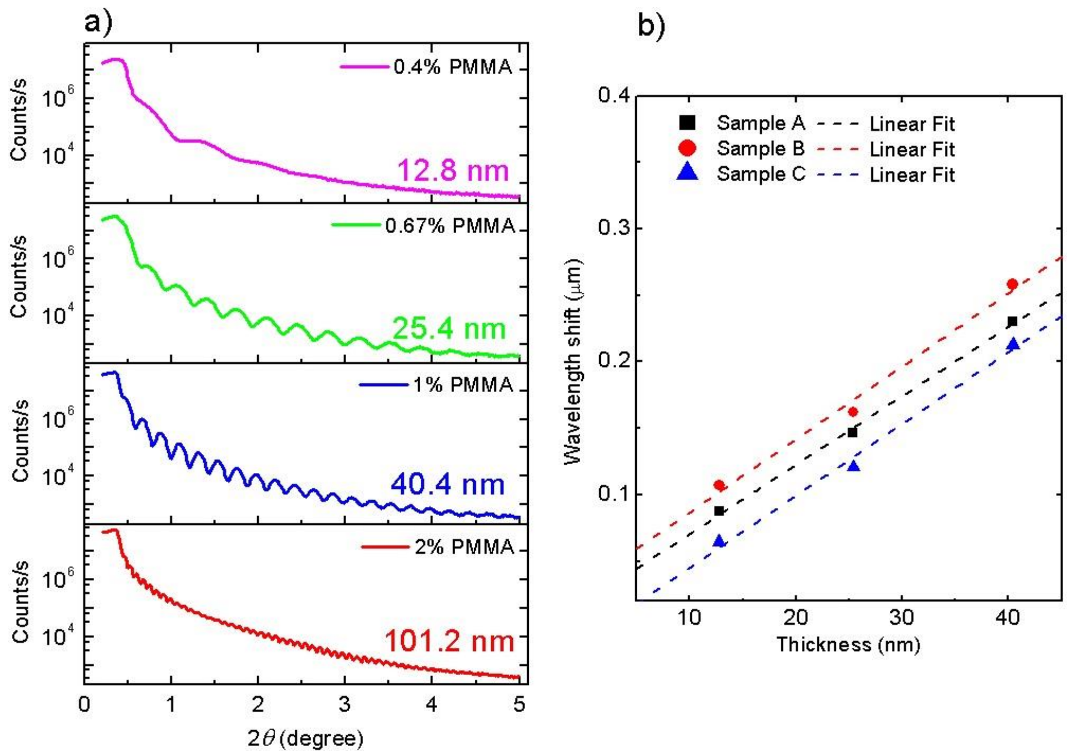

Figure S1. (a) X-ray reflectivity versus two times of incidence angle (angle between the incident beam and surface plane). The thicknesses of diluted polymers with different concentrations (marked in each plot) are determined through the oscillation periods observed in the X-ray reflectivity measurement. (b) Wavelength shift of Fano resonance versus the measured PMMA layer thickness.

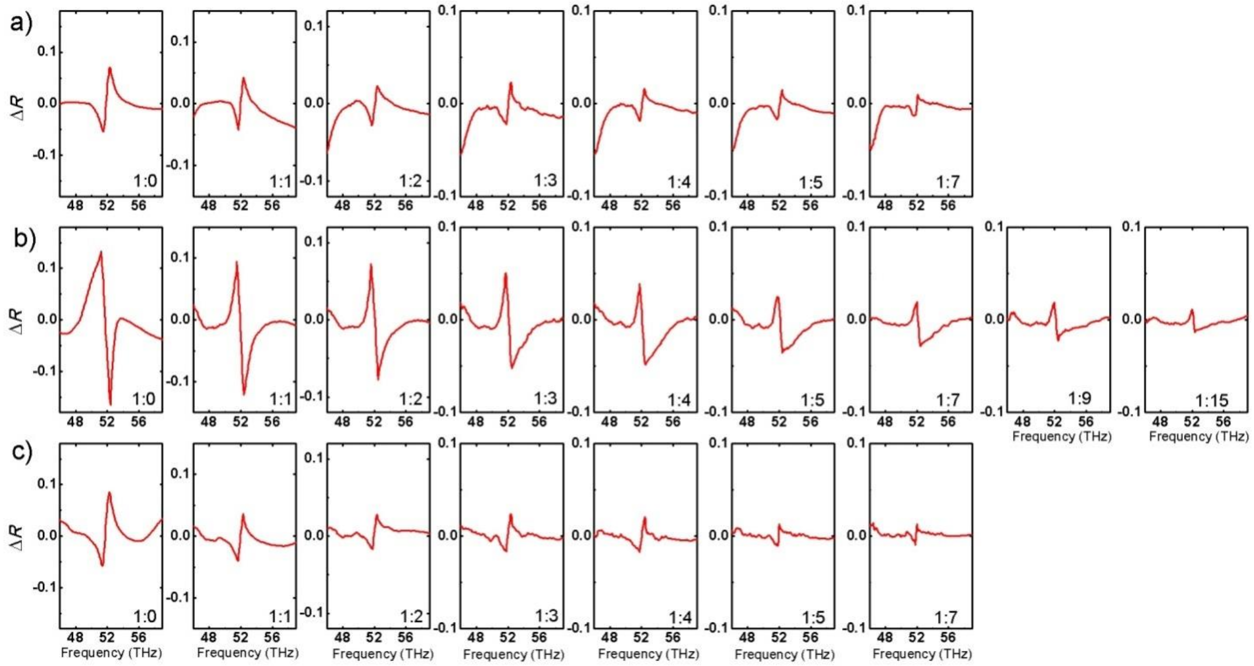

Figure S2. Reflectance difference spectra ΔR of sample A (a), sample B (b) and sample C (c) around the carbonyl bond absorption band, coated with dilute PMMA solutions. The volume ratio between PMMA resist (2% solid content in anisole) and pure anisole solvent are marked in each plot. Strongly enhanced absorption signal strengths are observed for sample B (on-resonance) as compared to sample A and sample C (off-resonance). Especially, when the volume ratio between anisole and PMMA resist is larger than 7, the absorption signal well above the noise level is observable only from sample B.

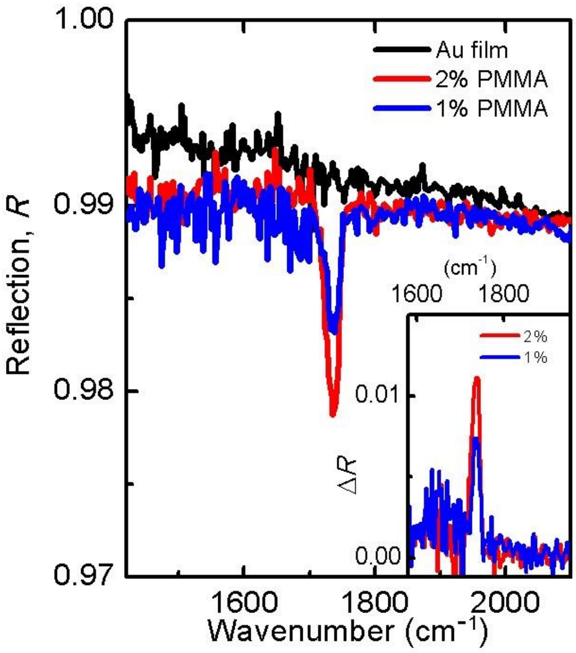

Figure S3. Reflection of the Au film reference substrate before (black) and after (red: 2% solid content, blue: 1% solid content) the coating of PMMA layer. The inset shows the reflectance difference spectra Δ*R* of the PMMA-coated gold films.

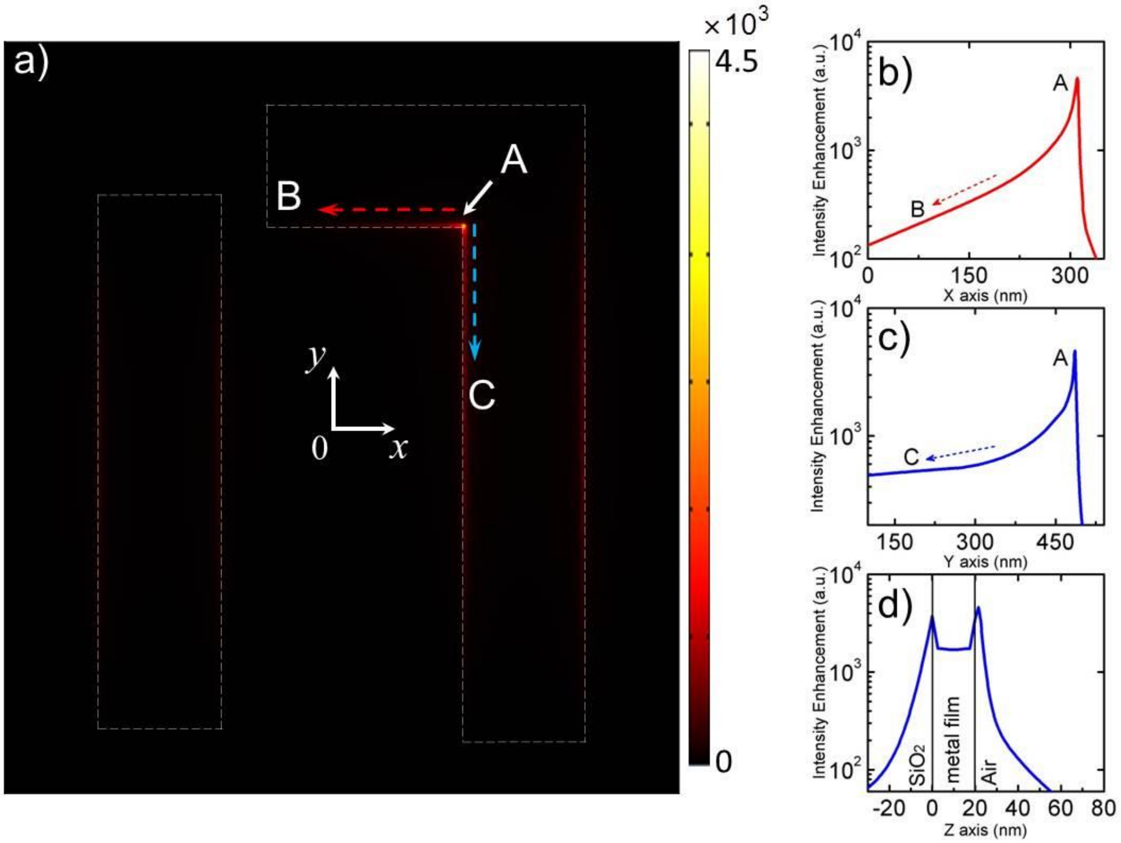

Figure S4. (a) Near-field profile and enhancement of electric field intensity $|\boldsymbol{E}|^2$ (a.u.) at Fano resonance (1 nm above the metasurface) for sample B. (b) Field profile variation along *x* direction from point A to illustrate the rapidly decaying field. (c) Field profile variation along *y* direction from point A. (d) Field profile variation along *z* direction at point A to show the evanescently decaying near-field distribution.

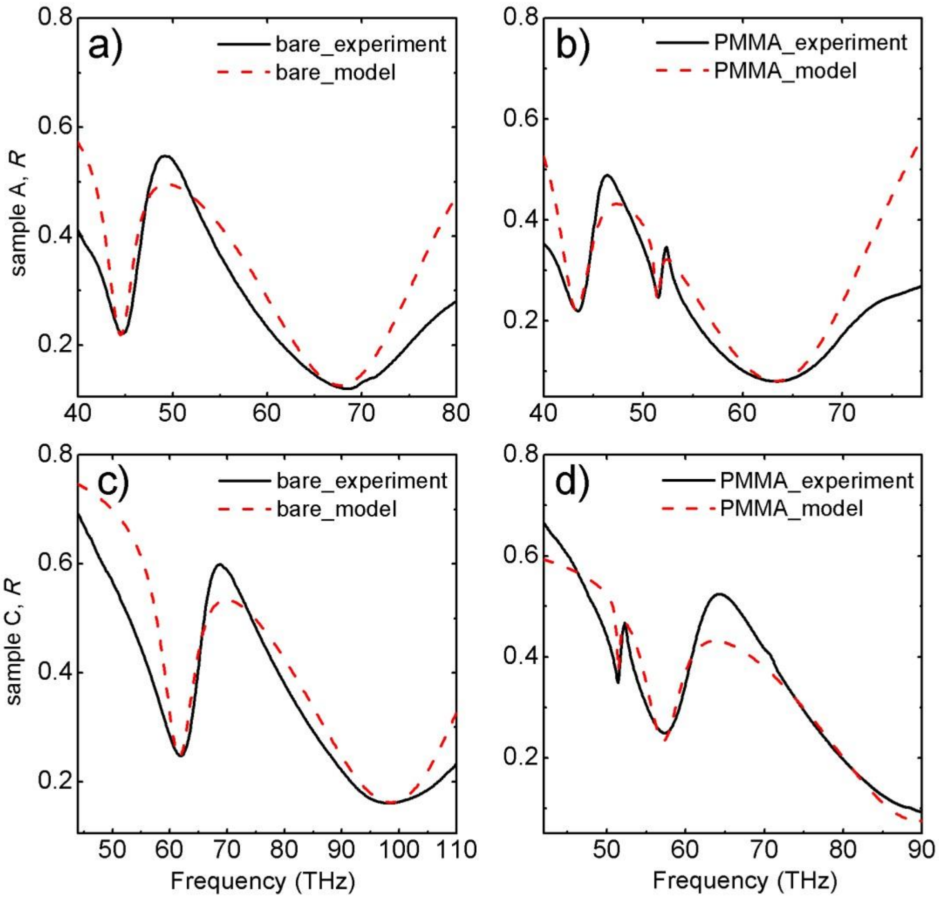

Figure S5. Comparison between the measured reflection spectra (black solid) and the calculated reflection spectra from the coupled harmonic oscillatormodel (red dashed) for both bare and functionalized sample A (a-b) and sample C (c-d).

Parameters	Sample A		Sample C	
	bare	PMMA	bare	PMMA
ω_d, γ_d	72.5, 34	66.8, 30.5	101, 36	93.5, 38
ω_q, γ_q	44.7, 3.8	43.4, 3.8	62, 5.2	57.4, 5.5
ω_m, γ_m		51.4, 0.8		51.5, 0.5
σ_{dq}	13.5	12.5	23	19
σ_{dm}		4.2		0
σ_{qm}		0		6.4

Table 1. Extracted fitting parameters used in the oscillator model for sample A and C: resonance frequencies (unit: THz), damping rates (unit: THz), and coupling strengths (unit: THz^2).

References

1. Joannopoulos, J. D., Johnson, S. G., Winn, J. N. & Meade, R. D. *Photonic Crystals: Molding the Flow of Light (Second Edition)*. (Princeton University Press, 2008).
2. Raman, A. & Fan, S. Perturbation theory for plasmonic modulation and sensing. *Physical Review B* **83**, 205131, doi:http://dx.doi.org/10.1103/PhysRevB.83.205131 (2011).
3. Antosiewicz, T. J., Apell, S. P., Claudio, V. & Käll, M. A simple model for the resonance shift of localized plasmons due to dielectric particle adhesion. *Opt. Express* **20**, 524-533, doi:10.1364/OE.20.000524 (2012).
4. Limaj, O. *et al.* Mid-Infrared Surface Plasmon Polariton Sensors Resonant with the Vibrational Modes of Phospholipid Layers. *The Journal of Physical Chemistry C* **117**, 19119-19126, doi:10.1021/jp402629g (2013).
5. Pucci, A. IR spectroscopy of adsorbates on ultrathin metal films. *physica status solidi (b)* **242**, 2704-2713, doi:10.1002/pssb.200541131 (2005).
6. Seo, E. K., Lee, J. W., Sung-Suh, H. M. & Sung, M. M. Atomic Layer Deposition of Titanium Oxide on Self-Assembled-Monolayer-Coated Gold. *Chemistry of Materials* **16**, 1878-1883, doi:10.1021/cm035140x (2004).
7. Le, F. *et al.* Metallic Nanoparticle Arrays: A Common Substrate for Both Surface-Enhanced Raman Scattering and Surface-Enhanced Infrared Absorption. *ACS Nano* **2**, 707-718, doi:10.1021/nn800047e (2008).
8. Pucci, A. *et al.* Surface enhanced infrared spectroscopy using gold nanoantennas. *physica status solidi (b)* **247**, 2071-2074, doi:10.1002/pssb.200983933 (2010).
